# Production of Stable Electrically Conductive PVDF Membranes Based on Polydopamine-Polyethyleneimine—Assisted Deposition of Carbon Nanotubes

**DOI:** 10.3390/membranes14040094

**Published:** 2024-04-20

**Authors:** Abdelrahman M. Awad, Charles-François de Lannoy

**Affiliations:** 1 Department of Chemical Engineering, McMaster University, 1280 Main Street West, Hamilton, ON L8S 4L7, Canada; awada9@mcmaster.ca; 2 Department of Chemistry and Chemical Biology, McMaster University, 1280 Main Street West, Hamilton, ON L8S 4L7, Canada

**Keywords:** PDA/PEI crosslinking, membrane stability, conductive membrane, PVDF, carbon nanotubes

## Abstract

Electrically conductive membranes (ECMs) have emerged as a multifunctional separation technology that integrates membrane filtration with electrochemical reactions. Physical stability remains a critical challenge for ECMs synthesized by coating polymer membranes with conductive materials. In this article, polydopamine (PDA) and polyethyleneimine (PEI) were used to facilitate the synthesis of significantly more stable ECMs using poly(vinylidene fluoride) (PVDF) ultrafiltration membranes and carbon nanotubes (CNTs). Four different synthesis methods were compared in terms of the final surface stability and separation properties: (1) CNTs deposited on PEI-crosslinked PDA-coated PVDF membranes, (2) PEI-crosslinked CNTs deposited on PDA-coated PVDF, (3) PDA, PEI and CNTs sequentially deposited layer-by-layer on PVDF, and (4) PEI-crosslinked PDA deposited on CNT-coated PVDF. The results revealed that method 1 generated ECMs with the greatest physical stability, highest electrical conductivity (18,518 S/m), and sufficient permeability (395.2 L/(m^2^·h·bar). In comparison, method 2 resulted in membranes with the highest permeability (2128.5 L/(m^2^·h·bar), but with low surface conductivity (502 S/m) and poor physical stability (i.e., 53–75% lower peel-off forces compared to other methods). Overall, methods 1, 3, and 4 can be used to make highly conductive membranes with a 97–99% removal of methyl orange by electrochemical degradation at −3 V.

## 1. Introduction

Electrically conductive membranes (ECMs) combine conventional membrane filtration with electrochemical reactions enabled by an applied electrical potential [1]. The electrochemical activity of ECMs has demonstrated a pivotal role in fouling control, rejection of charged solutes, and degradation of organic pollutants via reduction-oxidation (redox) reactions [2,3].

ECMs are primarily synthesized by conductive coating, where polymer membranes are coated with conductive materials (graphitic materials (e.g., carbon nanotubes (CNTs)) or metals) [4,5]. The physical stability of the conductive materials is one of the critical limitations of ECMs fabricated by the coating synthesis. Conductive nanoparticles can easily detach from the surface of ECMs during cross-flow water filtration, backflushing, or simply under long-term use [6]. Various approaches have been implemented to enhance the physical stability of ECMs. These methods encompass the use of binding agents (such as polyvinyl alcohol (PVA) [6,7] and polymethylmethacrylate) [8], microwave irradiation [9], hot pressing transfer [10], and post-deposition treatments with nitric acid [11]. While these techniques have demonstrated success in enhancing the membrane stability of ECMs made from specific polymers such as poly(ether sulfone) (PES) [12], cellulose nitrate [13], and poly(ethylene terephthalate) [11], no methods have been demonstrated to synthesize poly(vinylidene fluoride) (PVDF) conductive membranes with sufficiently high physical stability. Hence, a pressing necessity exists to develop new binding agents that can produce stable ECMs based on PVDF membranes. In this regard, polydopamine (PDA) is a promising binding molecule that can adhere to various organic and inorganic substances [14,15,16]. Polyethyleneimine (PEI) can be used to crosslink PDA to reduce the aggregate sizes coated on the substrate [17,18,19], or it can be used to crosslink CNTs which enhances nanoparticle dispersion on the membrane supports [20]. Although the PDA/PEI chemistry can potentially enhance the stability of PVDF ECMs, it is unclear how the coating methods impact the membrane permeability, electrical conductivity, and electrochemical activity.

It is hypothesized that different coating methods can impact the physical stability, membrane morphology, and separation properties of ECMs. This work examines four separate coating methods to prepare conductive PVDF membranes. The rationale for choosing the four methods is summarized in Table 1. In method 1, PEI and PDA were cross-linked together to assess the impact of PEI on PDA aggregates on the water permeability. Method 2 investigates the impact of PEI crosslinked- CNT dispersion on water permeability and electrical conductivity. Method 3 was used as a baseline where PDA, PEI, and CNTs were deposited independently on PVDF membranes. In method 4, we hypothesized that the deposition of PEI-crosslinked PDA on CNT-coated PVDF can enhance the water permeability of sable membranes. It is worth mentioning that PDA can be used to crosslink CNTs, however, our preliminary screening experiments revealed that the deposition of CNTs combined with PDA on PVDF resulted in membranes with extremely low conductivities (~sheet resistance was not measurable our eddy current device). Therefore, we excluded that coating approach to make ECMs in the current study. ECMs synthesized by the four methods were compared based on the membrane structure, permeability, surface conductivity, physical stability, and electrochemical activity.

## 2. Materials and Methods

### 2.1. Materials

PVDF ultrafiltration (UF) membranes (nominal pore size of 0.2 μm, molecular weight cutoff ~5000 KDa) were purchased from Sterlitech (Auburn, WA, USA). Carboxyl functionalized single-walled/double-walled CNTs (SW/DWCNTs) (outer diameter of 1–4 nm, length of 5–30 μm, purity > 90%, and functional content of 2.73 wt%) were purchased from Cheap Tubes Inc. (Grafton, MA, USA). All other chemicals were purchased from Millipore Sigma.

### 2.2. Membrane Preparation

**Figure 1:**
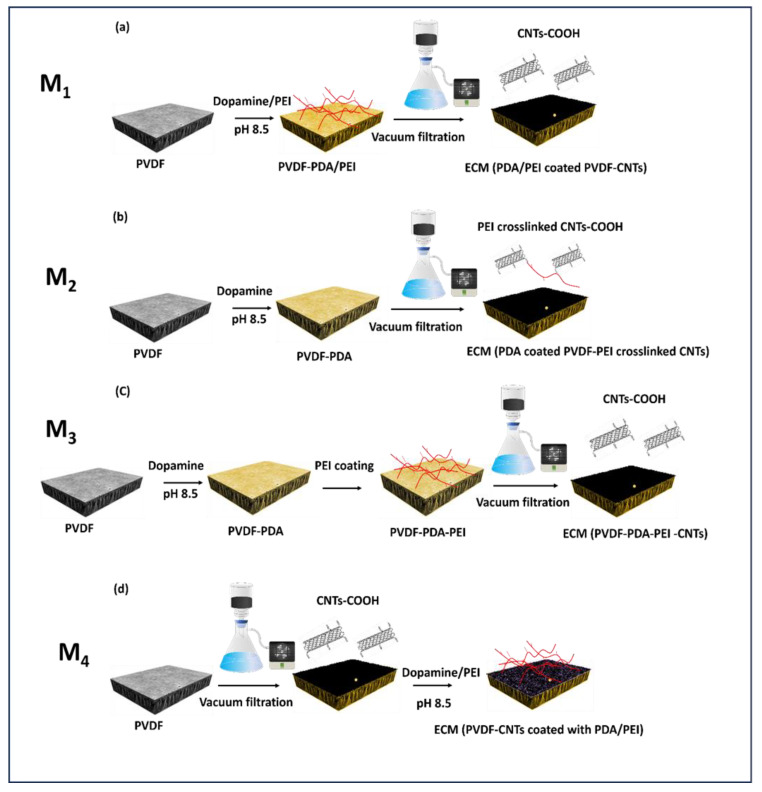
Schematic diagrams of synthesizing (**a**) M_1_, (**b**) M_2_, (**c**) M_3_, and (**d**) M_4_.

ECMs were prepared from UF PVDF membranes by four coating methods (Table 1, and Figure 1) as follows:

1. ***Method 1 (ECMs denoted as M_1_): CNTs were deposited on PEI crosslinked PDA-coated PVDF membranes:*** ECMs were prepared in a two-step process. In the first step, PVDF membranes were immersed (for 24 h) in a mixture of DA (2 mg/mL) and branched PEI (2 mg/mL, Mw 600) in a Tris buffer solution (0.1 mM, pH 8.5). In the second step, a previously prepared CNT suspension was deposited on the PDA-PEI-coated PVDF by vacuum deposition. To prepare the CNT suspension, 0.5 mg/mL CNT suspension in DI water was stirred for 5 min (600 rpm at ambient temperature) and ultrasonicated for 1 h (a microtip of 1/4″ diameter, intensity of 40%). with an interval of 2 s on and 2 s off. To facilitate the dispersion of CNTs, the suspension was mixed with 0.75 mg/mL solution of sodium dodecyl sulfate (SDS) in DI water previously stirred for 30 min (RPM 600, ambient temperature). The mixture of CNT and SDS suspensions was stirred for 15 min and ultrasonicated for 1 h at the same prob intensity and interval time as above. To deposit 5 mg of CNTs, 10 mL of 0.5 mg/mL CNT suspension was filtered under vacuum through wet PDA/PEI coated membranes placed in a glass flask under a pressure of 100 mbar.
2. ***Method 2 (ECMs denoted as M_2_): PEI crosslinked CNTs were deposited on PDA-coated PVDF membranes:*** ECMs were prepared in a two-step process, where PVDF membranes were first coated by PDA for 24 h (2 mg/mL DA in Tris buffer solution (0.1 mM, pH 8.5)), then PEI crosslinked CNTs were vacuum filtered through the PDA coated PVDF membranes. To prepare the PEI crosslinked CNTs, the carboxylic groups of CNTs were first activated by adding 25 mg of 1-Ethyl-3-(3-dimethylaminopropyl) carbodiimide (EDC) and 50 mg N-hydroxysuccinimide (NHS) into a 0.5 mg/mL CNT suspension. Then, 5 mL of 2 mg/mL branched polyethyleneimine (BPEI) (Mw 600) was added to the suspension and stirred for 24 h under ambient conditions to ensure a complete reaction between the amine group and the carboxylic groups of CNTs.
3. ***Method 3 (ECMs denoted as M_3_): PDA, PEI and CNTs were sequentially layer-by-layer deposited on PVDF membranes:*** ECMs were synthesized by a 3-step coating process. In the first step, PVDF membranes were coated with PDA (by immersion in DA 2 mg/mL in Tris buffer solution (0.1 mM, pH 8.5) for 24 h). In the second step, the membranes were dip-coated with a 2 mg/mL PEI solution for 12 h under ambient temperature. In step 3, a CNT suspension dispersed by 0.75 mg/mL SDS was vacuum filtered through the PDA-PEI coated membranes.
4. ***Method 4 (ECMs denoted as M_4_): PEI crosslinked PDA was deposited on CNT-coated PVDF membranes:*** In this method, a CNT suspension dispersed by 0.75 mg/mL SDS was first deposited onto pristine PVDF membranes, followed by membrane coating with PEI cross-linked PDA following the exact coating procedure explained in method 1.

Triplicate membranes were prepared for each method with a fixed CNT amount of 5 mg. A control membrane (denoted as control) was also prepared by deposition of CNTs on PVDF membranes without any modification. The fabricated membranes were dried under ambient conditions and stored in DI water before characterization and testing.

### 2.3. Membrane Characterization

The surface morphology was analyzed by field emission scanning electron microscopy (FE-SEM JEOL7000F). Prior to cross-sectional SEM imaging, the membranes were freeze-fractured with liquid nitrogen, and the samples were then dried completely and coated with 5 nm platinum. Surface wettability was studied by a highspeed contact angle instrument (OCA 35). Membrane sheet resistivity was measured using an eddy current device (EddyCus^®^ TF lab 2020 Series, SURAGUS, Dresden, Germany). The electrical conductivity of the membranes was estimated based on sheet resistance according to the following relationship:

(1) $$\alpha =\frac{l}{R}$$

where α is the electrical conductivity (S/m), R is sheet resistance Ohm/Sq, and l is the CNT thickness (m) estimated from SEM cross-sectional images.

Cyclic voltammetry (CV) curves were conducted to assess the reduction-oxidation reactions that can take place in the batch electrochemical cell with sodium chloride (NaCl) solution as the supporting electrolyte and methyl orange (MO) as a model contaminant. The CV tests were conducted between −3 V and 0 V at a scan rate of 10 mV/s using an Autolab Potentiostat device (Metrohm AG^®^ Instruments (Mississauga, ON, Canada). A peel-off (adhesion) test was performed using an adhesive Grand & Toy tape in an Instron 4411 tensile force tester. In brief, the tape was attached to 2 × 4 cm^2^ membranes previously fixed to a roller with double-sided tape. All membranes were fully dried before attaching the tape and air bubbles between the tape and membrane surface were removed by pressing. CNTs were then peeled off from the underlying support at a load cell of 0.05 kN and a crosshead speed of 2.5 mm/min. The force required to peel off the CNT layer was recorded by the series IX software where the adhesion force (N/mm) was estimated from the average force/membrane width. Wet physical adhesion of CNTs to membranes was also evaluated by immersing the ECMs in a bath sonication for an hour with a visual inspection of CNTs detachment from the membrane surface at different interval times of sonication.

The pure water permeability was measured in a dead-end stainless steel flow cell (Sterlitech, Auburn, WA, USA), as shown in Figure S1a. (more details can be found in supporting information (SI)). Electrochemical degradation experiments were conducted in a batch electrochemical cell, as shown in Figure S1b. (SI). A methyl orange (MO) aqueous solution with a concentration of 85 mg/mL was used as a contaminant model and NaCl solution as the supporting electrolyte (conductivity of ~5394 µS/cm). The conductive membranes (an effective surface area of 5.376 cm^2^) were used as the working electrode at an applied voltage of −3 V (i.e., cathode), while a graphite sheet (length of 42 mm of and width of 17 mm) was used as the counter electrode (i.e., anode). An autolab potentiostat/galvanostat was used to provide the required voltage for the electrochemical cell where the two electrodes were spaced 3 mm apart. The MO solution was continuously stirred at 700 rpm during the experiment, and 300 μL samples were collected from the mixture to measure the change in MO concentration with time. Dye concentrations in the collected samples were estimated based on the UV-vis absorbance at 464 nm (Tecan Spark 10 M UV–Vis Spectrophotometer), where the absorbance intensity was converted to MO concentration based on the calibration curve shown in Figure S2 of the supporting information.

## 3. Results and Discussion

### 3.1. Membrane Morphology

**Figure 2:**
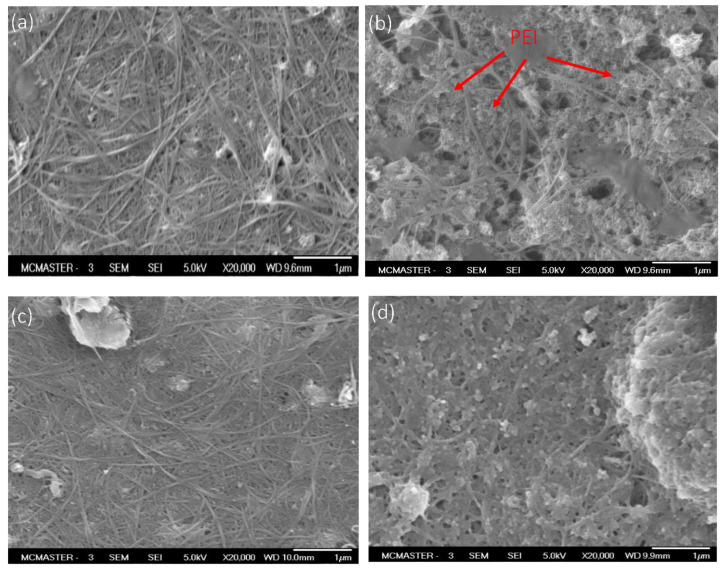
Surface SEM images (20 k magnification) of (**a**) M_1_, (**b**) M_2_, (**c**) M_3_, and (**d**) M_4_.

Surface SEM images of ECMs synthesized by the various methods are shown in Figure 2. Compared to their polymer membrane substrates (Figure S3), CNT-coated membranes had a significantly less porous structure, primarily due to pore blockage by deposition of the CNT nanoparticles. Figure 2. shows that ECMs prepared by methods 1 and 3 had similar surface morphology, with a structured CNT network on top of the support membranes. However, when PEI was used to cross-link the CNTs (i.e., M_2_), CNT nanoparticles were partially coated with PEI molecules, which resulted in the physical isolation of some CNTs (Figure 2b). Similar observations of the PEI coating of SWCNTs and MWCNTs were reported in the literature [20,21]. Figure 2d. reveals that ECMs synthesized by method 4 had a PDA/PEI coating on top of the CNT network. There were no distinguishable morphological differences in the cross-section SEM images of ECMs synthesized by the different methods as shown in Figure S4. Nonetheless, the ECM membrane thickness prepared by method 2 was slightly larger (4–4.5 vs. 2.5–3 µm) than those prepared by the other methods (M_1_, M_3_, and M_4_).

### 3.2. Water Permeability and Surface Conductivity

**Figure 3:**
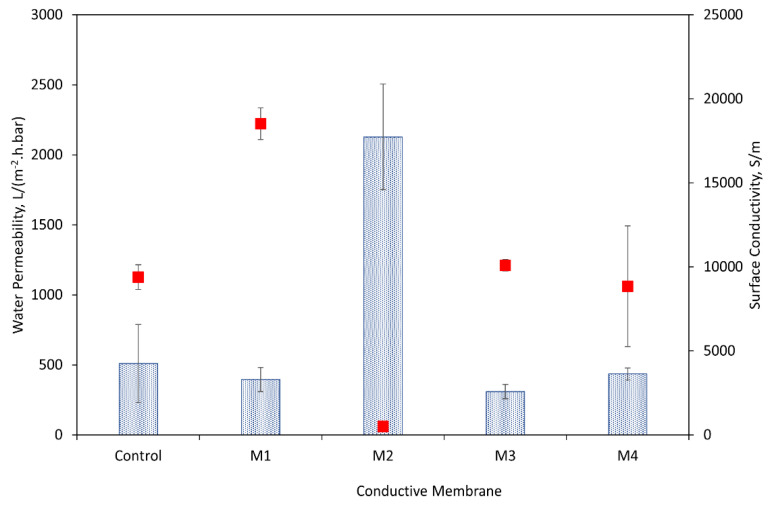
Pure water permeability (blue bar) and electrical conductivity (red squares, ¢) of control PVDF-CNT membrane, and ECMs synthesized by the four methods (M_1_–M_4_). The error bar is the standard deviation of triplicate samples.

The pure water permeabilities of ECMs are illustrated in Figure 3. Regardless of the coating method, ECMs exhibited substantial reductions in the water permeability (i.e., 61–93%) as compared to their underlying supports (Figure S5) as is common in the literature on graphene nanomaterial-based ECMs [4]. The lower permeabilities of ECMs are due to the increased flow resistance and the lower membrane porosity of the CNT coating (Figure 2 and Figure S3).

Figure 3 also reveals that the permeabilities of ECMs vary with the coating methods. Membranes prepared by method 2 demonstrated the greatest water permeability of 2128.5 L/(m^2^·h·bar), which was 81.4, 85.4, and 79.5% larger than the permeabilities of M_2_, M_3_, and M_4_, respectively. We hypothesize that the variation in the ECMs’ permeabilities was based on the membrane morphology and surface wettability. As discussed earlier, SEM images showed that in method 2, PEI molecules between CNTs can lead to a more open porous structure and less dense CNT coating as compared to the other coating methods (M_1_, M_3_, and M_4_). PEI molecules also play a role in membrane hydrophilization due to the presence of abundant hydrophilic amino groups. The contact angle (CA) measurements (Figure S6) of M_2_ (i.e., ECMs made by method 2) were found to be lower than the CA of ECMs prepared by the other methods. Dynamic descending CA was measured instead of static CA because the membranes have porous structures leading to the inevitable penetration of water droplets due to capillary effects. The more rapid decline rate in CA for M_2_ (~1.36°/second) as compared to that for M_1_ (~0.76°/second) and M_3_ (~0.54°/second) confirms both the more porous and hydrophilic coating of M_2_. ECMs prepared by method 4 also showed CA values lower than those observed for M_1_ and M_3_ indicating a more hydrophilic surface of M_4_ due to the existence of PDA/PEI hydrophilic molecules on top of the CNT layer.

Figure 3 reveals that method 1 can produce highly conductive membranes, with a conductivity being up to two orders of magnitude higher (18,518 vs. 502 S/m) than that of ECMs prepared by method 2. The lower conductivity observed for method 2 was attributed to the PEI coating of CNTs which led to the physical isolation of CNTs from each other and electrical insulation of individual CNTs which can prevent electron mobility between CNT nanoparticles. A similar observation of a reduction in the surface conductivity was reported by Duan et al. when CNTs were functionalized with PEI [22]. For ECMs made by method 4, only a slight reduction in the surface conductivity was recorded (8849 vs. 9389 S/m) as compared to the control PVDF-CNTs which implies that PDA/PEI post-coating has a limited impact on electron mobility in the CNT networks.

### 3.3. Membrane Physical Stability

**Figure 4:**
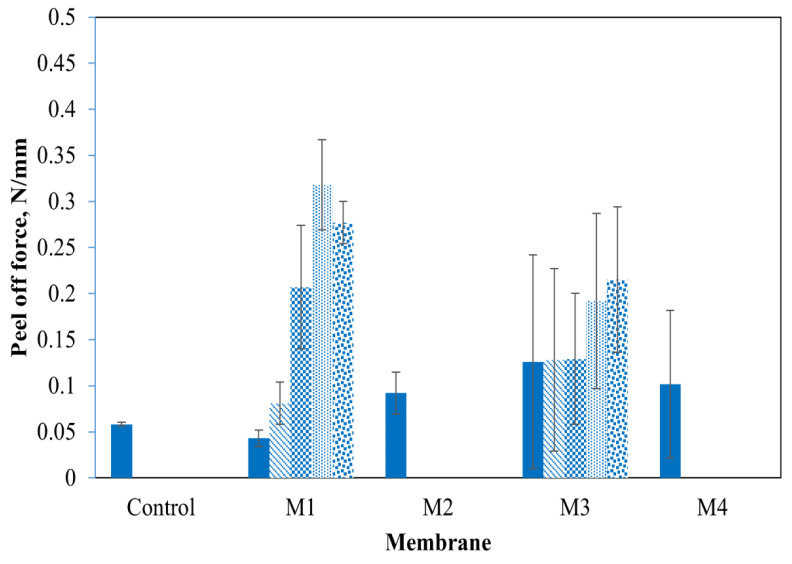
Peel-off forces for multiple consecutive tests (test 1 (solid bar), test 2 (stripped), test 3 (checkerboard), test 4 (dotted bar), and test 5 (confetti bar) of the control PVDF-CNT membranes and ECMs.

**Figure 5:**
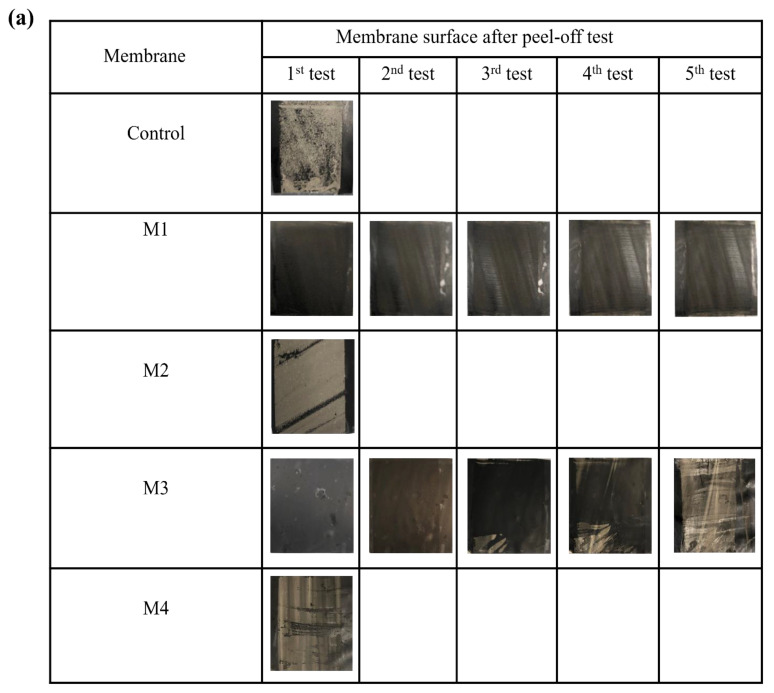

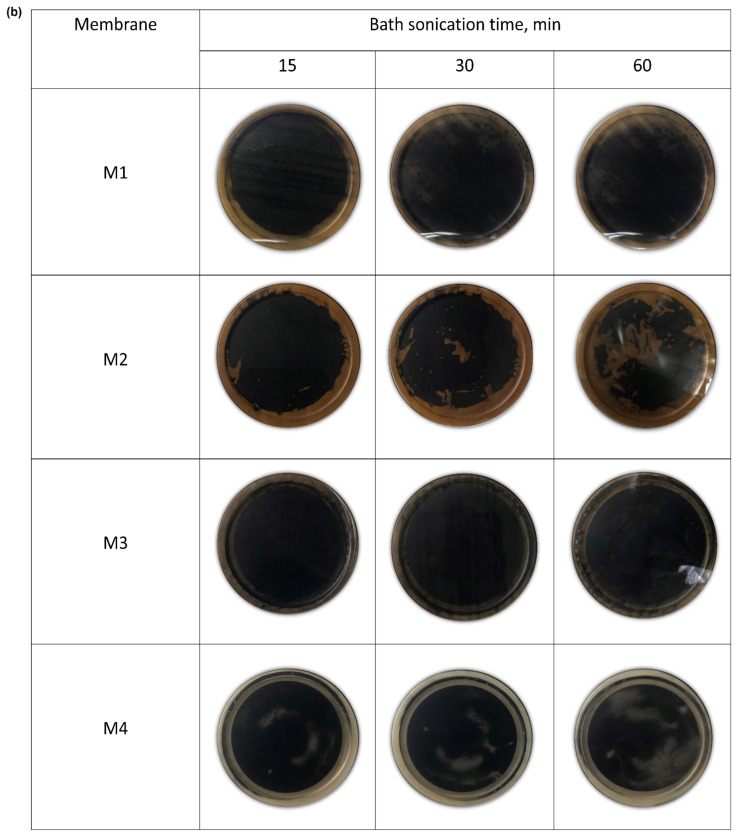
(**a**) Membrane surface after the peel-off tests for the control PVDF-CNT membrane and ECMs (**b**) physical stability of ECMs at different sonication elapsed times.

Dry adhesion experiments can quantify the forces needed to detach CNTs from substrate surfaces (i.e., ECM physical stability), as might occur under crossflow filtration or backflushing. Peel-off tests demonstrated that the physical stability of ECMs varies with the membrane coating methods. Figure 4 shows the recorded average peel-off forces, and the membrane surfaces after peeling off the CNTs are shown in Figure 5a. For M_2_ and M_4_, the majority of CNTs were removed from the membrane surface in a single peel-off test at a relatively small force (0.092 and 0.101 N·mm^−1^ for M_2_ and M_4_, respectively). Therefore, ECMs prepared by methods 2 and 4 have physical stability comparable to that of the control PVDF-CNT membrane (CNTs were removed in a single peel-off trial at a force of 0.058 N·mm^−1^). In contrast, five peel-off trials at significantly larger forces were needed to remove CNTs from M_1_ and M_3_. After the fifth peel-off trial, few CNTs were observed on the tape indicating that the adhesion forces between residual CNTs and polymer supports are greater than those between the tape and the CNTs. Among the tested ECMs, M_1_ demonstrated the highest physical stability as indicated by the greatest force of the last peel-off trial (i.e., 8.29 N or ~0.377 N/mm over a 22 mm strip of tape). Figure 4. also shows that the forces recorded for the first two trials are greater in M_3_ as compared to M_1_, which indicates a stronger CNT-CNT cohesion force in M_3_. This stronger cohesion force can be explained by the existence of a PEI layer on PDA-coated PVDF which leads to CNT crosslinking. Nevertheless, the force of the fifth peel-off test in M_1_ was higher than that in M_3_ indicating that PEI crosslinked PDA forms a stronger binder of CNTs and PVDF compared to PEI coated PDA (as in M_3_). Figure 4 reveals that after five peel-off tests, the majority of CNTs were removed in M_3_ (at a force of 0.215) while more residual CNTs were observed in M_1_ (at a force of 0.377), which indicates the better stability of ECMs made by M_1_. It is also worth noting that in M_4_ (i.e., post-coating method), the PEI crosslinked PDA can bind strongly to both the tape and CNTs, which resulted in the removal of significant amounts of CNTs in a single peel-off trial. We hypothesize that in M_4_, some PDA molecules can leach from the coating solution through the CNT layer to the PVDF surface, which can explain the comparable adhesive forces recorded for M_4_ (i.e., 0.1016 N/mm) and M_2_ (i.e., 0.092 N/mm).

The results of the bath sonication tests were consistent with the peel-off tests (Figure 5b). Overall, ECMs synthesized by methods 1 and 3 appeared to be more stable than ECMs prepared by methods 2 and 4. For M_2_ and M_4_, the membrane surfaces were severely damaged in 30 min sonication time, while in M_1_ and M_3_ the membranes were more stable for nearly 60 min.

### 3.4. Electrochemical Activity

**Figure 6:**
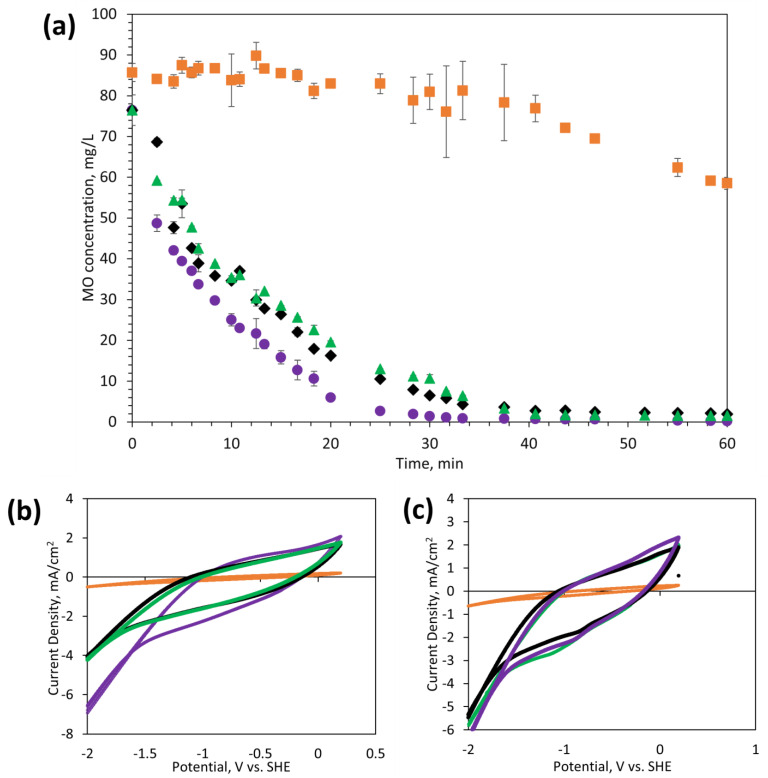
Batch removal of MO with: (**a**) M_1_ (purple circles, ●), M_2_ (orange squares, ■), M_3_ (black diamonds, **◊**), and M_4_ (green triangles, ▲) at a potential of −3 V, (**b**) cyclic voltammetry with NaCl supporting electrolyte (50 Mm) for M_1_ (purple line), M_2_ (orange line), M_3_ (black line), and M_4_ (green line); and (**c**) cyclic voltammetry wit MO solution for M_1_ (purple line), M_2_ (orange line), M_3_ (black line), and M_4_ (green line).

The electrochemical activity for redox reactions was assessed with MO as a model organic contaminant. Dye degradation experiments were conducted in a batch electrochemical cell using 85 mg/L MO and 1 M NaCl as a supporting electrolyte. ECMs were used as the working electrode (cathode) at an applied voltage of −3 V, while a graphite sheet was used as the counter electrode (anode). Figure 6a. demonstrates that the electrochemical activity of ECMs varies with their fabrication methods. Notably, M_2_ had significantly lower activity towards degradation of MO as compared to other ECMs (M_1_, M_3_, and M_4_), which can be ascribed to the lower electrical conductivity of M_2_ (Figure 3). Only 31.7% MO removal was observed with M_2_ within 60 min as compared to 99.6, 97.4, and 97.9 % removal with M_1_, M_3_, and M_4_. The degradation rate appears to be faster with M_1_ (1.27 mg/(mL^−1^·min^−1^) as compared to M_3_ and M_4_ (1.24 mg·mL^−1^·min^−1^); however, all three membranes demonstrated effectiveness in degrading organic contaminants (removal >97% in 60 min). Control experiments (Figure S7) were conducted at 0 V (i.e., no applied voltage) showing that ECMs without applied potential can remove only 10–14% of the MO by physical adsorption. MO removal at −3 V was, therefore, primarily due to electrochemically induced degradation.

To understand the degradation mechanisms, CV curves were conducted from 0 to −3 V in the NaCl supporting electrolyte alone (Figure 6b) and in MO solution with the supporting electrolyte (Figure 6c). For M_1_, M_3_, and M_4_, CV curves showed a prominent peak for direct reduction of MO at 0.90–1.08 V w.r.t SHE, and no peak was observed for M_2_. The peak at 0.90–1.08 V w.r.t SHE implies that MO degradation occurred by direct reduction of the MO at the cathode due to electron transfer. Dye degradation can also occur by indirect oxidation of MO mediated by the generation of oxidizing reagents (i.e., hydrogen peroxide) from two-electron oxygen reduction reactions. However, no oxidation peaks were observed in the CV curves, which indicates that indirect oxidation of MO may require a higher concentration of dissolved oxygen to generate sufficient hydrogen peroxide. Figure 6b,c also show that the current densities in MO/NaCl and NaCl alone were considerably higher for M_1_, M_3_, and M_4_ as compared to current densities recorded with M_2_, which confirms that the poor MO degradation in M_2_ was caused by its low surface conductivity.

## 4. Conclusions

Four different coating methods were examined to make PVDF conductive membranes by PDA/PEI-assisted deposition of CNTs on commercial UF PVDF membranes. The following conclusions can be drawn from the results:

- Membrane structures can be controlled by coating chemistry. Dense and stable conductive membranes can be made simply by adhering non-crosslinked CNTs to PDA/PEI-coated substrates. In contrast, open porous but unstable CNT structures can be obtained through PEI crosslinking of CNTs adhered to PDA. The presence of exposed PEI at the membrane surface leads to enhanced surface hydrophilicity, but less stable CNT coating. ECMs with exposed PEI showed significantly less physical stability (i.e., adhesion forces of 0.092–0.1016 N/mm) as compared to other ECMs synthesized in this study (adhesion forces of 0.215–0.377 N/mm).
- ECMs prepared by the different methods showed variations in the water permeability and electrical conductivity, which were attributed to the membrane surface structures and hydrophilicity. ECMs prepared by deposition of PEI crosslinked CNTs resulted in more permeable but less conductive membranes as compared to the other methods.
- The use of PEI crosslinked PDA as an intermediate layer between CNTs and PVDF demonstrated the most physically stable ECMs, as confirmed by both dry peel-off (adhesion force of 0.377 N/mm) and wet adhesion tests under bath sonication.
- Membranes with high electrical conductivities can facilitate the electrochemical degradation of MO organic contaminants. The MO degradation mechanism was primarily attributed to direct reduction at the membrane surface.

## Figures and Tables

**Table 1:** Summary of the Four ECMs Fabrication Methods.

Membrane	Rationale	Process Steps
M_1_	PEI and PDA were cross-linked together to assess the impact of PEI on PDA aggregates.	Step 1: PVDF coated with PEI cross-linked-PDA Step 2: CNT coating
M_2_	CNTs and PEI were cross-linked to assess the impact of PEI on CNT dispersion.	Step 1: PVDF coated with PDA Step 2: CNTs cross-linked with PEI Step 3: PEI cross-linked CNTs coating
M_3_	Each coating was deposited independently as a baseline. PEI is coated on PDA and crosslinked to CNTs	Step 1: PVDF coated with PDA Step 2: PVDF-PDA coated with PEI Step 3: CNT coating
M_4_	The order of PEI-PDA cross-linked coating was the inverse of M1, to assess the impact on CNT stability.	Step 1: PVDF coated with CNTs Step 2: PEI crosslinked PDA coating

## Data Availability

The original contributions presented in the study are included in the article/Supplementary Materials, further inquiries can be directed to the corresponding author.

## Supplementary Materials

The following supporting information can be downloaded at: https://www.mdpi.com/article/10.3390/membranes14040094/s1, Figure S1. Schematic diagram of (a) the permeability test set-up, and (b) the electrochemical MO degradation cell. Figure S2. Methyl orange concentration vs. UV-vis absorbance intensity at 464 nm. The calibrated follows a linear relation of y = 15.6x, with R^2^ of 0.9986. Figure S3. Surface SEM images of (a) PVDF, (b) PDA-coated PVDF, and (c) PDA/PEI-coated PVDF membranes. Figure S4. Surface SEM image of ECMs prepared by method 2. Figure S5. Cross-sectional SEM images of (a) M_1_, (b) M_2_, (c) M_3_, and (d) M_4_. Figure S6. Water permeability of underlying membrane supports. Figure S7. Contact angle measurements of M_1_ (green rhombus), M_2_ (purple circles) M_3_ (red squares), and M_4_ (black triangles). Figure S8. MO removal by only physical adsorption for M_1_ (purple circles, ●)), M_2_ (orange squares, ■), M_3_ (black diamonds, **◊**), and M_4_ (green triangles, ▲).
