# Supplementary material for: Production of Stable Electrically Conductive PVDF Membranes Based on Polydopamine-Polyethyleneimine—Assisted Deposition of Carbon Nanotubes
Source: Membranes (Basel). 2024 Apr 20;14(4):94. doi: 10.3390/membranes14040094 (PMC11052225; doi:10.3390/membranes14040094)
Supplement: Supplementary file 1 [file membranes-14-00094-s001.zip › membranes-2919761-supplementary.pdf]

Supporting Information

Production of Stable Electrically Conductive PVDF Membranes Based on Polydopamine-Polyethyleneimine Assisted Deposition of Carbon Nanotubes

Abdelrahman M Awad [1], Charles-François de Lannoy [1, 2, *]

[1] Department of Chemical Engineering, McMaster University, 1280 Main Street West, Hamilton, Ontario, L8S 4L7, Canada.

[2] Department of Chemistry and Chemical Biology, McMaster University, 1280 Main Street West, Hamilton, Ontario, L8S 4L7, Canada.

[*]Corresponding author: delannoc@mcmaster.ca

1. Permeability set-up and electrochemical MO degradation cell

The pure water permeability set-up is shown in Figure S1a. Prior to permeability measurements, the membranes were compressed under a pressure of 80-100 psi for 60 min. Pure water flux was then measured from the volume of the permeate water (measured by a digital balance (VWR, USA). The flux was measured at a transmembrane pressure of 10, 20, 30, and 40 psi. The water permeability (in litre/(m^2.h. bar) was estimated from the slope of the linear plot of flux vs transmembrane pressure.

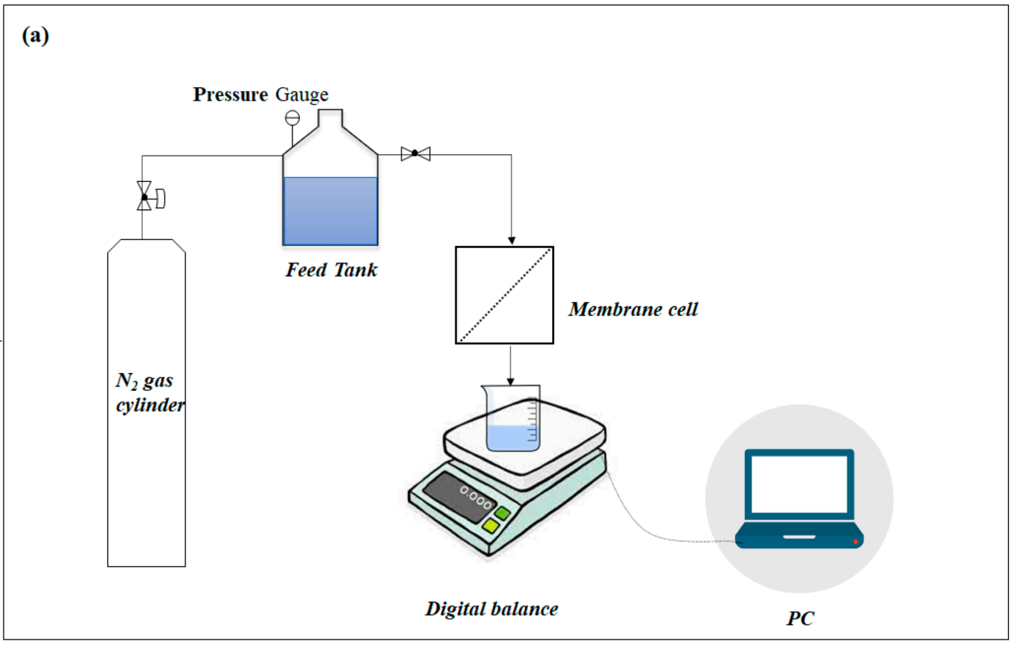

Supporting Information

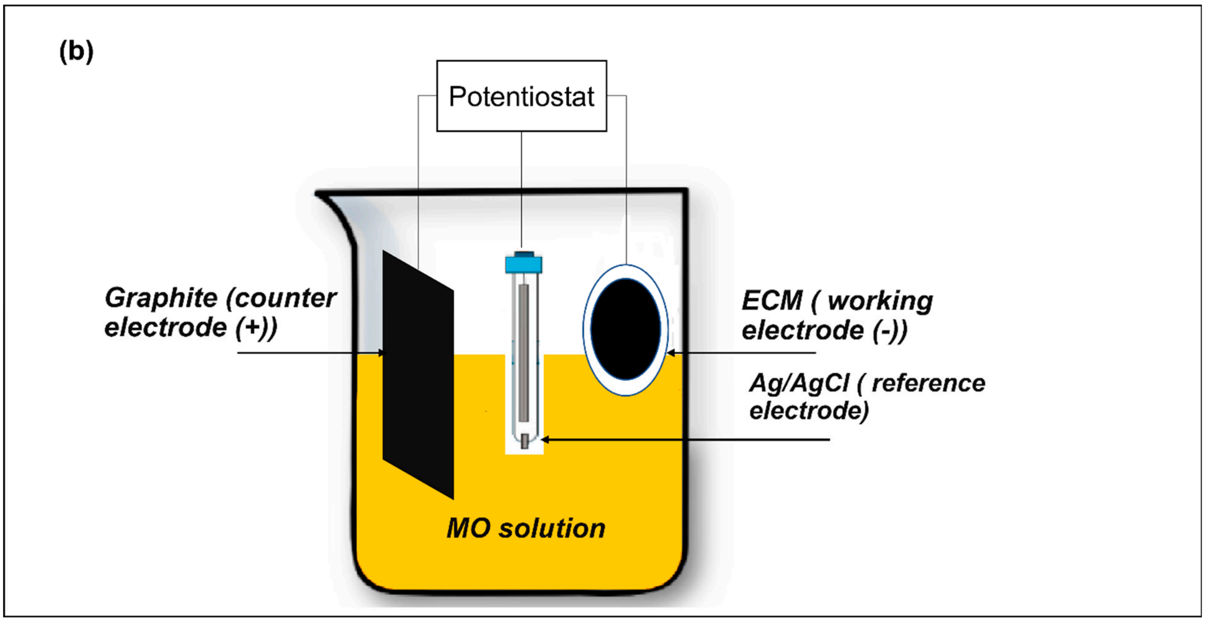

Figure S1. Schematic diagram of (a) the permeability test set-up, and (b) the electrochemical MO degradation cell.

2. Methyl Orange (MO) calibration curve

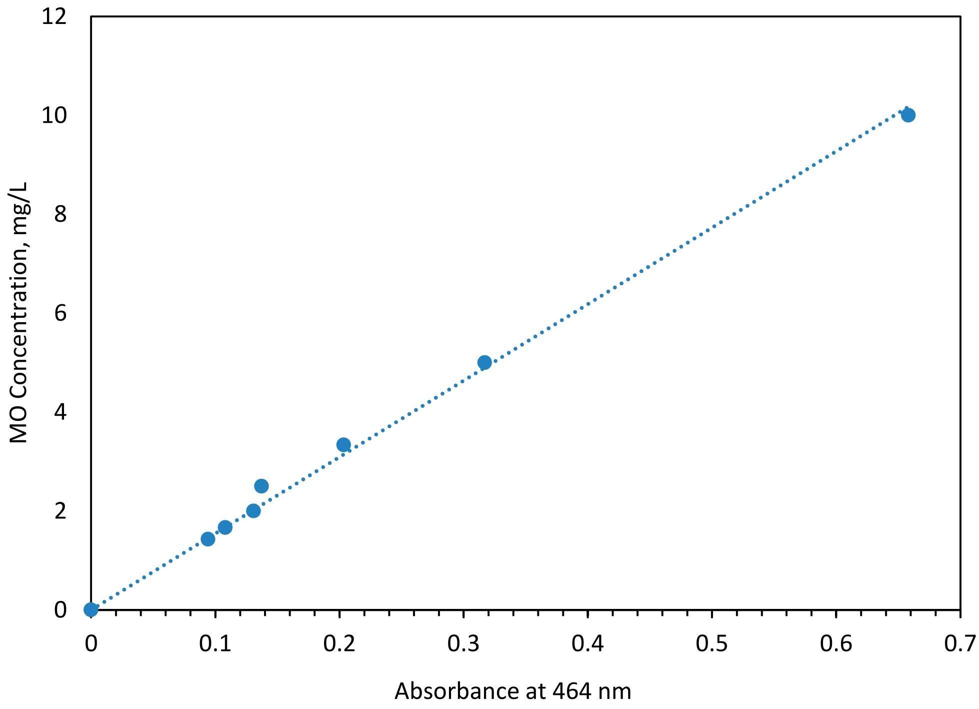

Figure S2. Methyl orange concentration vs UV-vis absorbance intensity at 464 nm. The calibrated follows a linear relation of y=15.6 x, with R^2 of 0.9986.

Supporting Information

3. *Surface Scanning Electron Microscope*

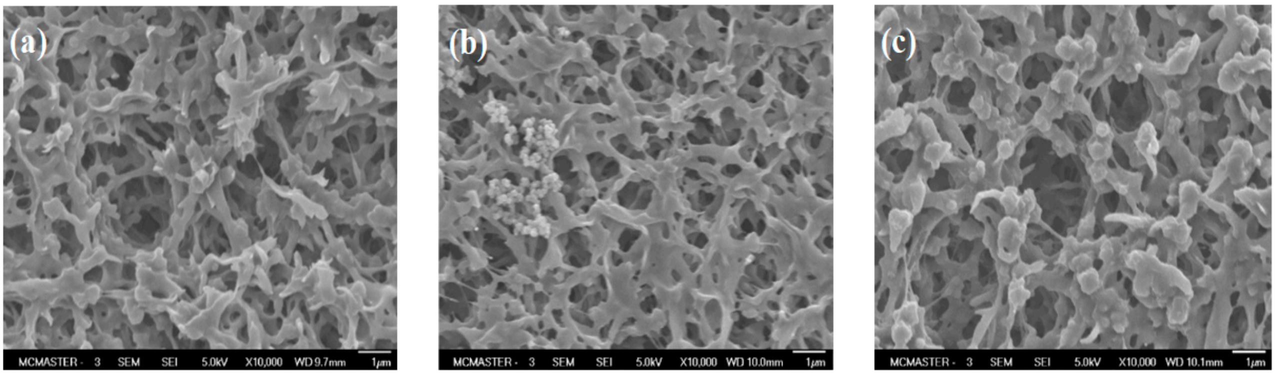

Figure S3. Surface SEM images of (a) PVDF, (b) PDA-coated PVDF, and (c) PDA/PEI-coated PVDF membranes.

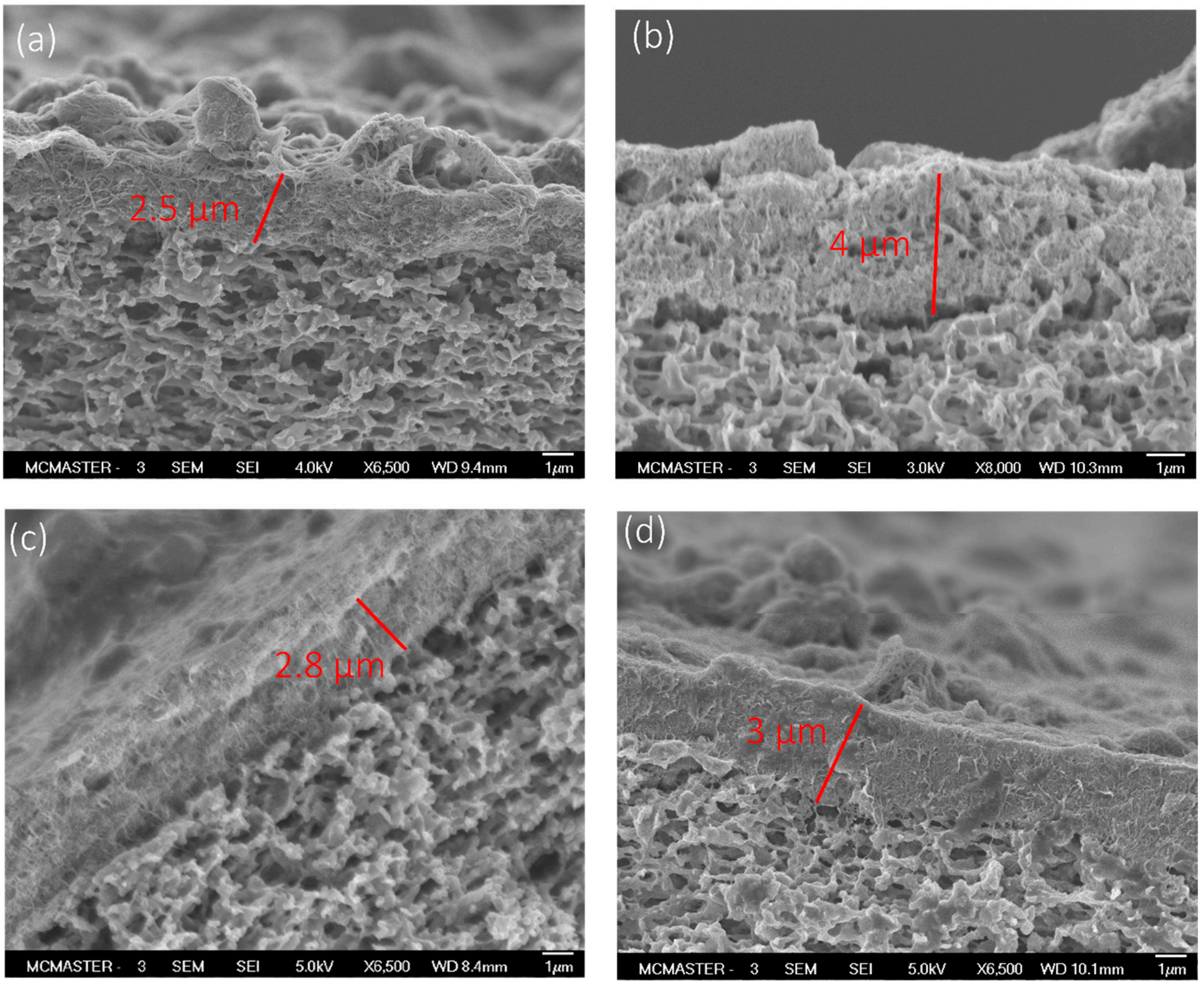

Figure S4. Cross sectional SEM images of (a) M_1, (b) M_2, (c) M_3, and (d) M_4.

4. Water permeability of membrane supports.

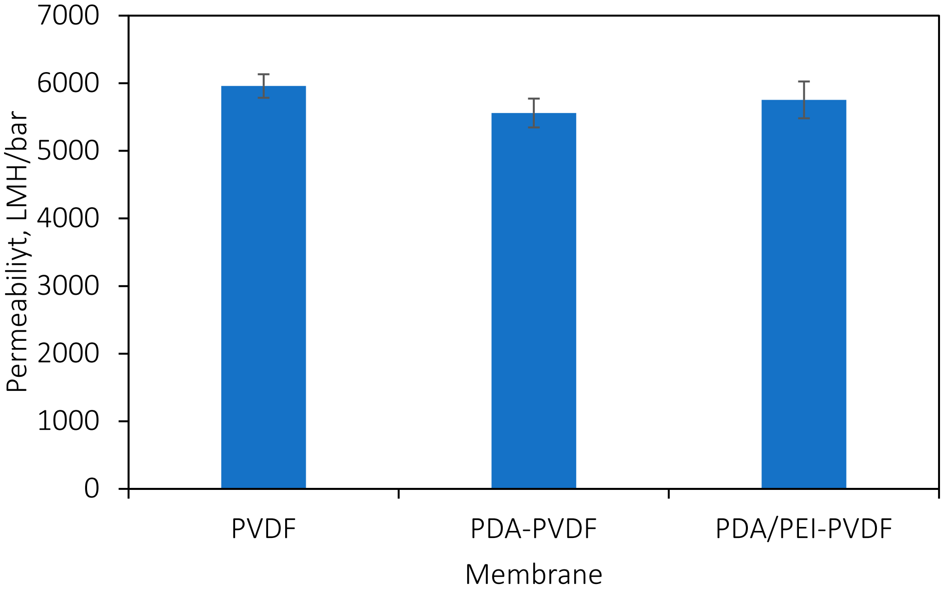

Figure S5. Water permeability of underlying membrane supports. ▲), data are mean values and error bars are standard deviations from 3 replicates

5. Contact angle measurements

The surface wettability of ECMs were evaluated based on contact angle measurements as shown in Figure S6. Dynamic CA was measured instead of static CA because the membranes have porous structures which leads to inevitable penetration of water droplets due to capillary effects. Even though the initial contact angles of membrane prepared by different methods were almost similar (~55±5.3°), the decline rate in the estimated CA was more rapid for ECMs produced by method 2. This lower CA can be explained by the fact that the hydrophobic CNTs are coated with the hydrophilic PEI as confirmed by SEM images.

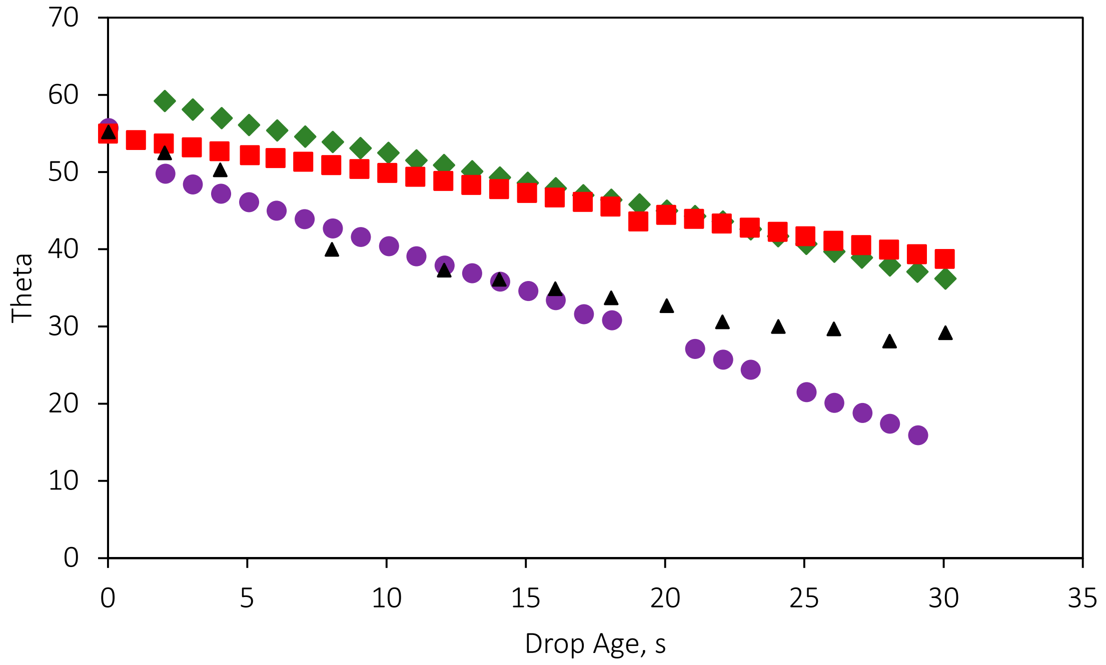

Figure S6. Contact angle measurements of M_1 (green rhombus), M_2 (purple circles) M_3 (red squares), and M_4. (black triangles).

5.. MO removal by physical adsorption

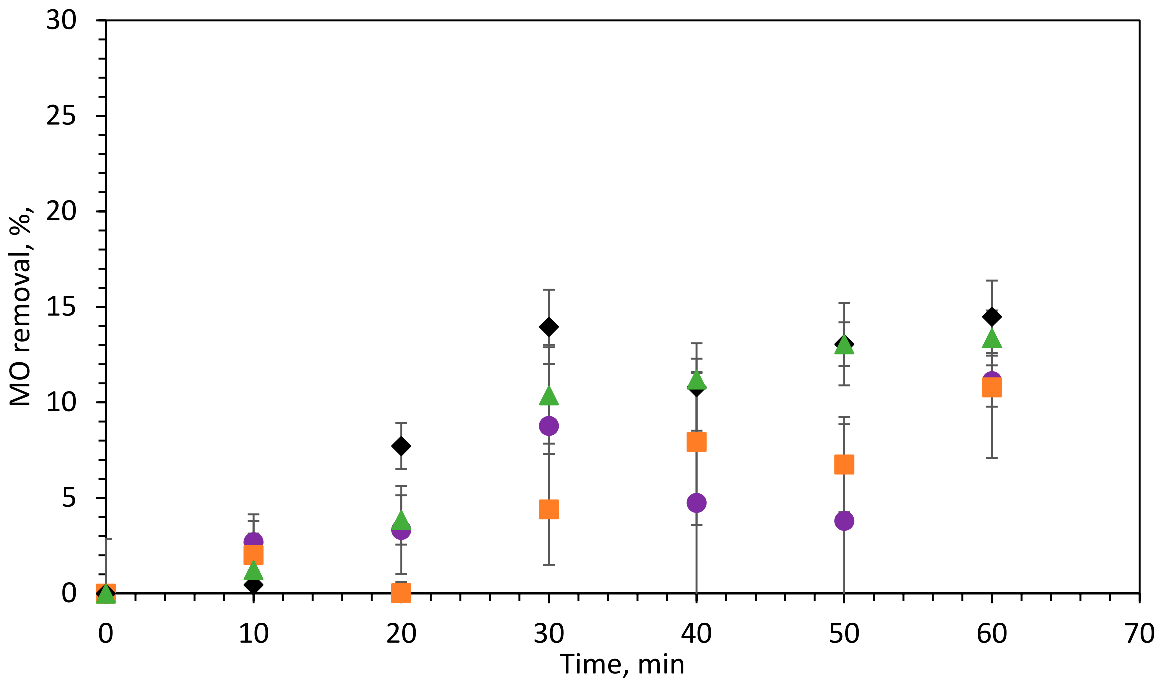

Figure S7. MO removal by only physical adsorption for M_1(purple circles, ●)), M_2 (orange squares, ■), M_3 (black diamonds, ♦), and M_4 (green triangles, ▲). Data are mean values and error bars are standard deviations from 3 replicates.

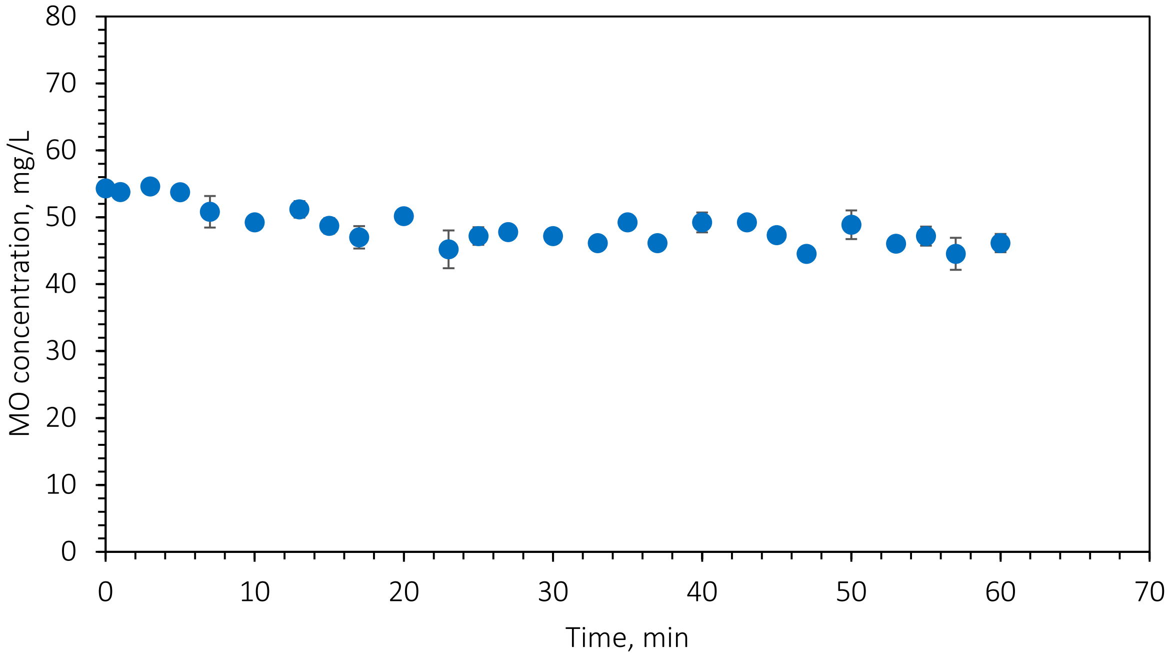

Figure S8. MO removal by PDA/PEI coated PVDPF control membrane as at -3Va applied potential.
